# Next-Generation Hedgehog/GLI Pathway Inhibitors for Cancer Therapy

**DOI:** 10.3390/cancers11040538

**Published:** 2019-04-15

**Authors:** Elisabeth Peer, Suzana Tesanovic, Fritz Aberger

**Affiliations:** Department of Biosciences, Paris-Lodron University of Salzburg, Cancer Cluster Salzburg, Hellbrunner Strasse 34, 5020 Salzburg, Austria; elisabeth.peer@sbg.ac.at (E.P.); suzana.tesanovic@sbg.ac.at (S.T.)

**Keywords:** Hedgehog signaling, Smoothened inhibitors, drug resistance, GLI transcription factors, cancer stem cells

## Abstract

The Hedgehog/Glioma-associated oncogene homolog (HH/GLI) signaling pathway regulates self-renewal of rare and highly malignant cancer stem cells (CSC), which have been shown to account for the initiation and maintenance of tumor growth as well as for drug resistance, metastatic spread and relapse. Efficacious therapeutic approaches targeting CSC pathways, such as HH/GLI signaling in combination with chemo, radiation or immunotherapy are, therefore, of high medical need. Pharmacological inhibition of HH/GLI pathway activity represents a promising approach to eliminate malignant CSC. Clinically approved HH/GLI pathway inhibitors target the essential pathway effector Smoothened (SMO) with striking therapeutic efficacy in skin and brain cancer patients. However, multiple genetic and molecular mechanisms resulting in de novo and acquired resistance to SMO inhibitors pose major limitations to anti-HH/GLI therapies and, thus, the eradication of CSC. In this review, we summarize reasons for clinical failure of SMO inhibitors, including mechanisms caused by genetic alterations in HH pathway effectors or triggered by additional oncogenic signals activating GLI transcription factors in a noncanonical manner. We then discuss emerging novel and rationale-based approaches to overcome SMO-inhibitor resistance, focusing on pharmacological perturbations of enzymatic modifiers of GLI activity and on compounds either directly targeting oncogenic GLI factors or interfering with synergistic crosstalk signals known to boost the oncogenicity of HH/GLI signaling.

## 1. Cancer Stem Cells and Hedgehog/GLI Signal Transduction

Cancer development is the consequence of an accumulation of genetic and epigenetic alterations that endow mutated cells with an unrestricted proliferative and eventually metastatic phenotype [1,2]. Although malignant cells are generally of clonal origin, deep genome sequencing in combination with functional assays interrogating the tumorigenic potential of individual cancer cells have revealed a surprising heterogeneity within the tumor mass. Of note, the capacity of clonal cancer cells to initiate, propagate, metastatically spread and resist drug treatment differs considerably within the same tumor mass. During the past years, numerous studies have provided convincing evidence that these highly aggressive phenotypes are present in rare and self-renewing cancer cells with stem-like cell characteristics that are likely to derive from long-lived tissue stem cells. These rare yet highly malignant cells are commonly referred to as cancer stem cells (CSC) with the ability to initiate, maintain and propagate heterogeneous malignancies [1,3,4,5,6,7,8]. Given the critical role of CSC in malignant development, the identification and selective targeting of CSC pathways such as Hedgehog, Wnt, Notch, Hippo and Janus Kinase/Signal Transducer and Activator of Transcription (JAK/STAT) signaling is of high medical need. Despite the availability of highly efficient drugs, e.g., inhibiting oncogenic HH/GLI signaling, a priori and acquired drug resistance as well as severe side effects pose major hurdles to the successful eradication of CSC. In this review, we focus on HH/GLI signaling as a driver signal in CSC and summarize the various molecular mechanisms conferring resistance to approved HH/GLI pathway inhibitors. In light of several recent findings about alternative pathway activation mechanisms, we discuss novel and promising approaches relying on the selective targeting of downstream HH pathway components and modifiers to inhibit the oncogenic activity of GLI transcription factors.

## 2. Canonical HH/GLI Signaling

The Hedgehog (HH) signaling pathway, which was originally discovered in the fruit fly by the Nobel prize laureates Nüsslein-Vollhard and Wieschaus in 1980 [9], controls a variety of processes during embryonic development as well as in tissue homeostasis, regeneration and healing in adults, explaining its oncogenic effect where aberrant activation of HH/GLI signaling causes or promotes various human malignancies.

**Figure 1:**
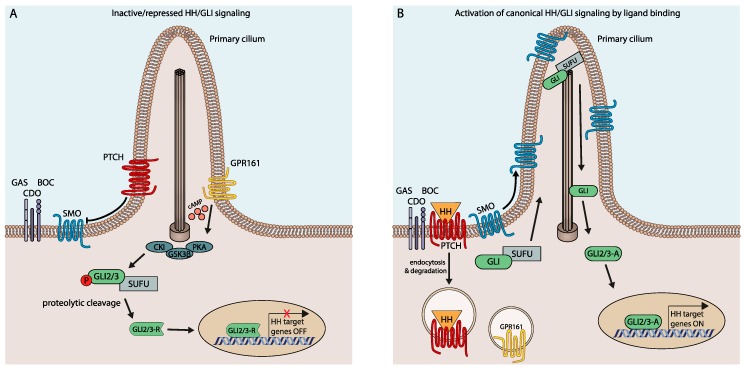
Canonical SMO-dependent HH/GLI signaling. (**A**) Pathway repression and GLI repressor formation in the absence of HH ligand protein. Unliganded PTCH1 prevents the ciliary translocation of the pathway effector SMO. GRP161 contributes to pathway silencing by increasing cAMP levels and PKA activity, resulting in enhanced GLI repressor formation. (**B**) Binding of HH ligand protein to its receptor PTCH triggers endocytic internalization of PTCH, thereby allowing ciliary entry and activation of SMO. Ciliary SMO attenuates PKA activity, thereby promoting the formation of unprocessed GLI activator forms and transcriptional activation of HH/GLI target genes, respectively.

In vertebrates, HH/GLI signaling is coordinated within the primary cilium and involves a highly complex regulatory relay mechanism with numerous effectors and modifiers (for extensive reviews, see References [10,11,12,13,14,15,16,17,18,19]). Notably, in the absence of ligand, the pathway is actively repressed by the unliganded form of the twelve-transmembrane domain receptor Patched (PTCH1), thereby preventing the activation and ciliary entry of the essential pathway effector Smoothened (SMO), a seven-transmembrane protein with similarities to G protein-coupled receptors [20,21] (Figure 1A). Intriguingly, the repressive activity of PTCH1 and its negative impact on SMO activation involves PTCH1-mediated reduction of cholesterol availability in the inner leaflet of the cell membrane [22].

PTCH1-mediated repression of SMO via cholesterol deprivation allows the sequential phosphorylation of the glioma-associated oncogene homolog transcription factors GLI1/2/3 by PKA, casein kinase 1 alpha (CK1α) and glycogen synthase kinase-3 (GSK3) [23], a process that is supported by the GLI-binding protein and negative HH pathway regulator Suppressor of Fused (SUFU) [24,25,26]. Phosphorylation of GLI promotes the binding of E3 ubiquitin ligases, subsequently leading to proteolytic cleavage and processing of the GLI2/3 transcription factors into the N-terminal transcriptional repressor form (GLI-R) (reviewed by References [15,27]) (Figure 1A).

HH/GLI pathway activation is triggered by binding of HH ligands (Sonic (SHH), Indian (IHH) or Desert Hedgehog (DHH)) to PTCH1, which blocks cholesterol removal activity and causes endosomal internalization of PTCH1. Ligand-mediated PTCH1 inactivation allows the phosphorylation of SMO at its C-terminus by CK1α and G protein-coupled receptor kinase 2 (GRK2) [28] and enhances cholesterol/oxysterol loading of SMO, thereby promoting its translocation into the primary cilium and its activation [29,30,31,32,33]. Activated SMO potently prevents the proteolytic processing of GLI repressor forms, resulting in high-levels of GLI activator proteins that translocate to the nucleus to induce expression of HH target genes (Figure 1B), including the third GLI family member GLI1, which further amplifies the HH signal response at the transcriptional level [15,16,26]. 

In mammalian HH/GLI signaling, the three GLI transcription factors contribute with varying degrees to the activator and repressor forms, depending on their C-terminal activation and N-terminal repressor domains. GLI1 lacks the repressor domain and therefore acts exclusively as an activator, while in the absence of active HH signaling, GLI3 and to a lesser extent GLI2, can be cleaved into repressor forms containing the N-terminal repressor and DNA binding domain but lacking the C-terminal transactivation cassettes [26,34,35]. Thus, it is the ratio of GLI activator and repressor forms that determines HH signal strength and target gene levels [15,16]. In addition to repressor formation, GLI activity is also negatively regulated by degron sequences promoting the continuous degradation of GLI proteins via the proteasomal machinery [36]. Depending on the signal receiving cell type, HH/GLI can activate distinct target gene sets that mediate various cellular activities, many of which are aberrantly activated in cancer settings such as cell proliferation, survival, migration and epithelial-to-mesenchymal transition (EMT) [19]. Fine tuning of signal strength and duration also includes the upregulation of positive and repressive pathway members such as GLI1, whose activation amplifies signaling, or PTCH1 and the HH interacting protein (HHIP), which both attenuate signaling [18,37,38].

## 3. Oncogenic HH/GLI Signaling

Several of the diverse cellular events regulated by HH/GLI relate to the hallmarks of cancer summarized by Hanahan and Weinberg [1,39], for instance, the control of proliferation, survival, angiogenesis, migration and cellular metabolism [19,40,41,42]. In line with these regulatory properties of HH/GLI, uncontrolled and persistent HH/GLI activity has been causally implicated in the growth of various cancer entities, including malignancies of the skin, brain, lung, prostate, breast, the gastrointestinal and hematopoietic system [43,44,45,46,47,48,49,50,51,52,53,54,55,56,57,58].

Much of our present understanding of oncogenic HH/GLI signaling stems from the genetic and molecular analysis of basal cell carcinoma (BCC), a very frequent nonmelanoma skin cancer, and medulloblastoma (MB) development [59,60]. The vast majority of BCC is caused by loss-of-function (LOF) mutations of *PTCH1* resulting in irreversible and ligand-independent pathway activation [46,61,62,63,64]. In addition to LOF mutations of *PTCH1*, gain-of-function mutations in *SMO* also account for sporadic BCC development [65,66,67]. Further, the SHH subgroup of MB displays constitutively active HH/GLI signaling, also as a consequence of *SUFU* LOF mutations, or genomic amplification of *GLI2* [68,69,70]. 

Apart from mutational activation of canonical HH/GLI signaling, several cancer entities with high medical need display noncanonical, SMO-independent mechanisms involving a number of prominent oncogenic players, e.g., RAS/MEK/ERK, PI3K/AKT, JAK/STAT, epigenetic modifiers or various members of distinct kinase families (for details, see below) that directly or indirectly impinge on and enhance GLI activity. SMO-independent activation of oncogenic GLI activity significantly enlarges the number of malignant entities with HH/GLI dependence and also accounts for resistance to clinically approved HH pathway inhibitors. A detailed understanding of the molecular signals and mechanisms conferring SMO-independent GLI activation in cancer cells is, therefore, of critical importance for the development of novel and efficacious drugs. Moreover, it would facilitate the exploration of treatment strategies that target the highly malignant yet rare CSC, where activated GLI proteins have been shown to be major mediators of their detrimental properties in disease progression, spread and drug resistance [15,16]. For instance, in human colon carcinoma, HH/GLI signaling not only correlates with the enhanced metastatic potential but also with self-renewal and activation of stemness genes [71]. Similarly, glioma stem cells (GSCs) rely on active HH/GLI signaling for their tumorigenic and clonogenic properties [72], as do highly malignant CSCs in pancreatic cancer, melanoma, leukemia and other aggressive entities with high medical need [73,74,75,76,77]. Targeting oncogenic HH/GLI activity in CSC alone or in combination with, for instance, chemo-, radio- or immune–therapeutic strategies is, therefore, a promising approach to improve the overall survival of many cancer patients by reducing cancer growth, metastatic spread, resistance development and relapses. 

## 4. Therapeutic Targeting of Oncogenic HH/GLI Signaling

The classical and clinically successful approach of targeting oncogenic HH/GLI signaling has mainly focused on the development of small molecules selectively inhibiting the essential HH effector SMO. Seminal work by the Beachy group has shown that cyclopamine, a naturally occurring steroidal alkaloid from the corn lily *Veratrum californicum*, potently inhibits HH/GLI signaling by binding to and blocking the activation of SMO [78]. Since then, several biotech and pharmaceutical companies have put significant efforts into developing clinically suitable SMO inhibitors (SMOi), of which three compounds referred to as vismodegib, sonidegib and glasdegib have so far been approved for the treatment of locally advanced and metastatic BCC (vismodegib and sonidegib) [64,79,80,81,82] and in combination with low-dose chemotherapy for acute myeloid leukemia (AML; glasdegib) [83,84,85].

Despite the striking therapeutic efficacy of SMOi, most notable in nonmelanoma skin cancer, there are a number of challenges and hurdles such as a priori and/or acquired drug resistance, severe adverse effects and limited response rates that need to be overcome in order to improve and expand the clinical application of HH/GLI pathway inhibitors.

In the following, we provide an overview of the different mechanisms of drug resistance, including SMO-independent regulation of oncogenic GLI proteins, followed by a more speculative chapter on how the current knowledge about resistance to HH pathway inhibitors can be overcome by single or rational combination targeting of HH effectors and GLI-promoting modifiers, including synergistic crosstalk signals amplifying the oncogenicity of HH/GLI signaling.

Despite the impressive success of SMOi in the treatment of nonmelanoma skin cancers, the benefit of SMO targeting in BCC patients is temporarily limited, with a median duration response of close to 8 months and a median progression-free survival of nearly 10 months [80,82]. In general, the response to SMOi apparently depends on the mutational rate of the tumor: Nevoid basal cell carcinoma syndrome (NBCCS) patients with *PTCH1* mutation but otherwise low mutational rate showed a 100% response rate without frequent and rapid drug resistance development during the study [86]. By contrast, only 43% of advanced BCC and 30% of metastatic BCC patients responded to SMO antagonist treatment [79]. Moreover, more than 20% of patients with advanced BCC and initial response to vismodegib treatment later acquired drug resistance, leading to relapse and tumor regrowth, respectively [87]. Apparently, this suggests that sporadic BCC with high mutational burden are more likely to develop resistance by the acquisition of additional mutations abolishing the therapeutic efficacy of SMOi before or during therapy.

## 5. Mechanisms of Drug Resistance in HH/GLI Targeting

### 5.1. Resistance Mutations in HH/GLI Pathway Components

**Figure 2:**
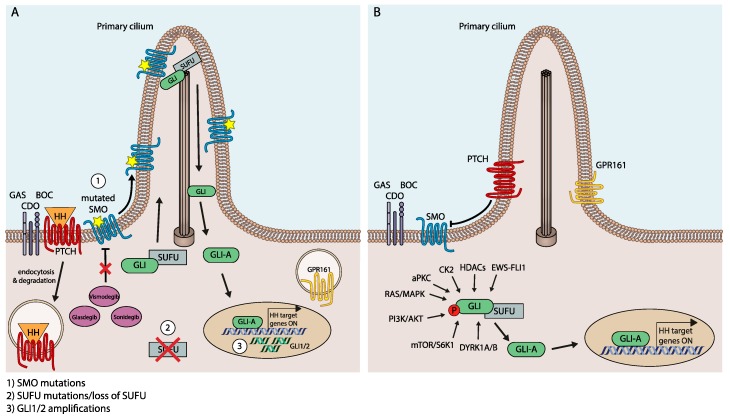
Genetic and molecular mechanisms conferring resistance to SMO inhibitors. (**A**) Three distinct mechanisms accounting for SMO inhibitor (SMOi) resistance are displayed: (1) Mutations in SMO itself, (2) genetic loss of downstream pathway repressors such as Suppressor of Fused (SUFU), and (3) genomic amplification of pathway effectors such as GLI2. (**B**) SMO-independent mechanisms of oncogenic GLI regulation contributing to the development of resistance to SMO inhibitors (for details, see main text).

First insight into the molecular and genetic mechanisms responsible for resistance to HH/GLI pathway inhibitors came from the genetic analysis of a medulloblastoma patient, who after an initial response to vismodegib treatment had relapsed and was deceased shortly thereafter [88]. Sequencing of the tumor DNA revealed a novel nonsynonymous SMO mutation replacing aspartic acid with histidine at amino acid position 473 (SMO^D473H^). In vitro assays demonstrated that SMO^D473H^ activated HH/GLI signaling to a comparable level as SMO^WT^ in the absence of PTCH1 activity. Structural modeling of SMO showed that Asp^473^ faces the highly conserved central binding cavity for GPCR modulators. Drug-target binding studies revealed that SMO^D473H^ has lost its high affinity binding to vismodegib, thereby explaining the patient′s relapse and the emergence of resistance to vismodegib [89] (see Figure 2A).

Missense mutations in *SMO* have also been identified as a cause for acquired drug resistance after continuous treatment of HH-induced murine medulloblastoma with sonidegib. Sequence analysis of genomic DNA from resistant tumors identified a total of five distinct *Smo* mutations (Smo^L225R^; Smo^N223D^; Smo^S391N^; Smo^D388N^; Smo^G457S^), which all significantly decreased the potency of sonidegib to block HH/GLI signaling [70] (Figure 2A).

A more systematic sequencing analysis of a large number of BCC patients resistant to vismodegib treatment has shed more light on the mutational landscape of SMO mutations in the context of resistance development [66,67,90]. The authors reported that 50% of SMOi-resistant patients displayed SMO mutations that maintained pathway activation upon treatment with SMOi. The sequencing results in combination with structure–function studies identified two distinct classes of SMO mutations conferring resistance to SMOi, i.e., alterations affecting the binding of inhibitor molecules and mutations resulting in constitutively active and inhibitor-resistant SMO variants lacking intrinsic auto-inhibitory activity. Intriguingly, resistance to SMO inhibitors could be overcome by targeting downstream components such as GLI2 and atypical protein kinase C (aPKC) [91,92] (see below for more details).

In addition to mutations in the SMO proto-oncogene, genetic alterations of HH/GLI pathway members acting downstream of SMO can contribute to SMOi resistance. Buonamici et al. found amplification of the *Gli2* gene in a large cohort of SMOi-resistant mouse MB. *Gli2* gene amplification correlated with mRNA expression, and *Gli2* knockdown in cells isolated from *Gli2*-amplified tumors partially reconstituted sensitivity to the SMOi sonidegib [70]. Furthermore, loss of the GLI repressor SUFU also confers resistance to SMO inhibitors, in line with findings showing that resistance to sonidegib treatment is paralleled by a reduction of SUFU protein levels and genomic loss [67,93,94] (Figure 2A).

### 5.2. SMOi Resistance via SMO-Independent Induction of Oncogenic GLI Activity

A priori resistance and limited response rates to SMOi treatment remain a major challenge to HH/GLI pathway targeting. Although multiple reasons may account for the lack of objective responses to SMOi in several clinical trials, we propose here that SMO-independent activation of oncogenic GLI activity represents an important and frequent cause for the often disappointing results in patients with cancers other than BCC, MB or AML. 

Signal crosstalk and integration between HH/GLI and other prominent oncogenic pathways plays a critical role in noncanonical, SMO-independent regulation of GLI activity. In several malignant entities, including melanoma, prostate cancer, pancreatic cancer, lung cancer and glioma, oncogenic RAS/MAPK signaling has been identified as an enhancer of transcriptional activation and function of GLI [93,95,96,97,98,99]. Based on in silico and in vitro screens, the ERK2 kinase can phosphorylate GLI1 at its N-terminus, leading to SMO-independent GLI protein stabilization and increased transcriptional activity [99]. In line with these findings, GLI1 is also activated via the MAPK cascade in lung adenocarcinoma, including the cancer stem cell compartment, via activation of KRAS or VEGF signaling [100]. Intriguingly, activation of RAS/MAPK signaling can also confer resistance to SMOi in BCC cells by promoting the transdifferentiation into a squamous cell carcinoma phenotype [93], a process that involves the loss of primary cilia in BCC cells anteceding the emergence of the MAPK-dependent SCC phenotype [92] (Figure 2B). 

SMO-independent GLI activation and thus resistance to SMOi can also be caused by integration with aberrantly activated PI3K/AKT signaling as well as its downstream effectors, such as S6K1 and atypical protein kinase C ι/λ (aPKCι/λ). Oncogenic GLI activation via PI3K/AKT signaling involves multiple mechanisms, such as enhancement of GLI protein stability by counteracting the negative regulatory role of PKA [101] and direct phosphorylation by S6K1, resulting in the disruption of the GLI-SUFU complex and GLI activation, respectively [102], and direct phosphorylation of GLI in its zinc finger domain, which enhances DNA binding and also confers resistance to SMOi [91]. The critical role of PI3K/AKT in SMO-independent activation of GLI is underlined by the finding that medulloblastoma cells resistant to SMOi display upregulation of PI3K/AKT signaling and sensitivity to PI3K targeting [70] (Figure 2B).

Another class of kinases regulating GLI activity in a SMO-independent manner is the family of dual-specificity tyrosine-(Y-) phosphorylation regulated kinases (DYRKs), which positively and negatively control HH/GLI signaling in a context-dependent manner. While DYRK2 has been shown to negatively regulate HH/GLI by promoting the degradation of GLI2 [103], DYRK1B can enhance GLI activity in an SMO-independent fashion. Genetic as well as pharmacological targeting of DYRK1B interferes with both SMO-dependent and SMO-independent GLI activation [94]. In pancreatic cancer cells, however, DYRK1B has been shown to act downstream of RAS repressing autocrine and favoring paracrine HH/GLI signaling and stromal GLI activation [104] (Figure 2B).

Like members of the DYRK family, casein kinases affect the activity of GLI proteins in both positive and negative ways. While CK1A directly phosphorylates GLI proteins to enhance GLI processing and degradation [23,105], CK2 has been shown to positively impact GLI activity by increasing protein stability [106] (Figure 2B).

Although phosphorylation of GLI proteins by a variety of kinases appears to be the dominant mechanism of SMO-independent GLI regulation [107], epigenetic writers, erasers and modifiers have recently been implicated in the regulation of oncogenic HH/GLI signaling. For instance, GLI acetylation represents a post-translational modification that negatively modulates GLI transcriptional activity, with histone acetylases accounting for the enzymatic transfer of repressive acetylation marks to GLI proteins. Conversely, histone deacetylases (HDACs) can remove the acetyl groups from GLI, leading to enhanced GLI transcriptional activity. Intriguingly, GLI1 is able to upregulate HDAC1 expression, thereby forming a positive feedback loop enhancing the level of deacetylated GLI activators [108,109]. SMO-independent activation of HH signaling also involves epigenetic modifiers, such as the bromo and extra C-terminal (BET) bromodomain 4 (BRD4). BRD4 is able to positively interact with and enhance the *GLI1* and *GLI2* promoters. Consistently, inhibition of BRD4 led to a reduction of GLI activity and tumor growth in both SMO-dependent and -independent cancer models [110] (Figure 2B).

Another promising and therapeutically relevant mechanism of SMO-independent GLI activation conferring SMOi resistance in BCC is mediated by the serum response factor (SRF)-megakaryoblastic leukemia 1 (MKL1) DNA-binding factors. SRF together with its co-activator (MKL1) binds adjacent to GLI target gene promoters and directly interacts with GLI1 to amplify GLI transcriptional activity. In addition to these mechanistic insights into the regulation of GLI activity, nuclear localization of MKL1 predicts SMOi resistance and responsiveness to MKL1 inhibitor treatment [111].

Furthermore, the oncogenic EWS-FLI1 transcription factor has been shown to activate HH signaling by directly inducing GLI1 expression, which plays an important role in the pathogenesis of Ewing Sarcoma [112,113,114]. 

### 5.3. Signal Crosstalk As Positive Modulator of Oncogenic HH/GLI Signaling and Mechanism of Drug Resistance

Apart from a number of post-translational modifications in response to the oncogenic cues outlined above, cell-intrinsic signal crosstalk and integration of HH/GLI with other cancer pathways also contribute to SMO-independent GLI regulation. Our own studies have shown that the epidermal growth factor (EGFR) pathway cooperates with HH/GLI in oncogenic transformation via activation of the RAS/RAF/MEK/ERK/JUN axis, where synergy is mediated via cooperative activation of HH–EGFR target gene promoters, a mechanism that involves selective and simultaneous promoter binding of GLI and JUN transcription factors [96,115,116]. Similarly, pro-inflammatory signals such as IL6 can cooperate with HH/GLI in promoting cancer growth via a molecular mechanism where GLI and the IL6/JAK2-activated transcription factor STAT3 bind and synergistically activate common HH-IL6 target genes [117]. In line with an important role of EGFR and IL6 signaling in GLI-driven cancers, genetic and pharmacological inhibition of EGFR and IL6/JAK2/STAT3 signaling, respectively, interferes with GLI-driven tumor growth [116,117].

Further insight into the molecular mechanisms of signal integration and drug resistance has come from the analysis of genetic mouse models of BCC that display at least partial resistance to SMOi treatment. These studies identified the developmental pathways Notch and Wnt as major players in the modulation of sensitivity to SMO targeting. Using an inducible BCC mouse model, Eberl et al. (2018) showed that the SMOi-sensitive suprabasal compartment was characterized by increased Notch signaling when compared to the SMOi-resistant basal compartment made up of palisading BCC cells. Notably, latent activation of Notch signaling was sufficient to induce regression of established BCC lesions, suggesting that pharmacological modulation of Notch signaling can improve the efficacy and overall response to SMOi [118].

Two recent studies about HH/GLI-driven BCC development and SMOi resistance development have identified quiescent cells positive for the stem cell marker *Lgr5* to survive the treatment with SMOi, leading to tumor relapse. This mechanism could be confirmed in human BCC, where patient biopsies taken before, during and after HH inhibitor therapy showed that GLI1 expression had nearly completely vanished, while small tumorigenic lesions expressing LGR5 persisted [119]. The importance of WNT signaling in SMOi resistance development was further underlined by a study of Biehs et al. showing in murine BCC models that SMOi treatment induced a cell identity switch from hair follicle bulge stem cells to stem cells of the intrafollicular epidermis (IFE) or isthmus (ISTH). Lineage-tracing experiments revealed that vismodegib treatment led to a reprogramming of transcriptional patterns in residual BCC cells with a strong activation of Wnt signaling from the beginning of vismodegib treatment. Together with the molecular analysis of human BCC samples, these finding suggest that during vismodegib treatment, residual surviving BCC cells can adopt a new, Wnt-driven identity that no longer relies on HH/GLI signaling and therefore become resistant to anti-HH therapy [120].

## 6. Overcoming SMO-Inhibitor Resistance by Next Generation HH/GLI Antagonists and Rational Combination Treatments

### 6.1. Targeting Drug Resistant SMO

The first set of next generation HH/GLI antagonists targeted SMO mutants insensitive to conventional SMO inhibitors, such as vismodegib. A screen for SMO antagonists that can inhibit SMO^D473H^ (or Smo^D477G^ in mouse) [89] found two compounds binding to distinct sites: ALLO-1 and ALLO-2 were able to inhibit Gli-luciferase expression in the presence of Smo^D477G^ with only a twofold difference compared to wild-type Smo, while vismodegib was less than 200-fold effective [121].

Although its further clinical development has been stopped due to failure in trials with pancreatic cancer patients, saridegib, a derivate of cyclopamine with improved potency and solubility [122], efficiently reduced HH-driven medulloblastoma and inhibited GLI1 expression even in the presence of the SMO^D473H^ resistance variant. Although mice that were long-term treated with saridegib did eventually develop drug resistance, this was not associated with detectable Smo mutations or amplifications of Hh pathway effectors, such as *Gli2.* Rather, resistance was caused through activation of P-glycoprotein (Pgp), an ATP-binding cassette (ABC) transporter, leading to increased drug efflux from cells. Indeed, additional therapy with the Pgp inhibitor verapamil could reverse drug resistance [123]. However, in a study with BCC patients that had developed resistance to vismodegib and were then treated with saridegib, nine out of 94 patients were unable to respond to saridegib, suggesting some overlapping resistance mechanisms for the two SMO inhibitors in human patients [124].

**Figure 3:**
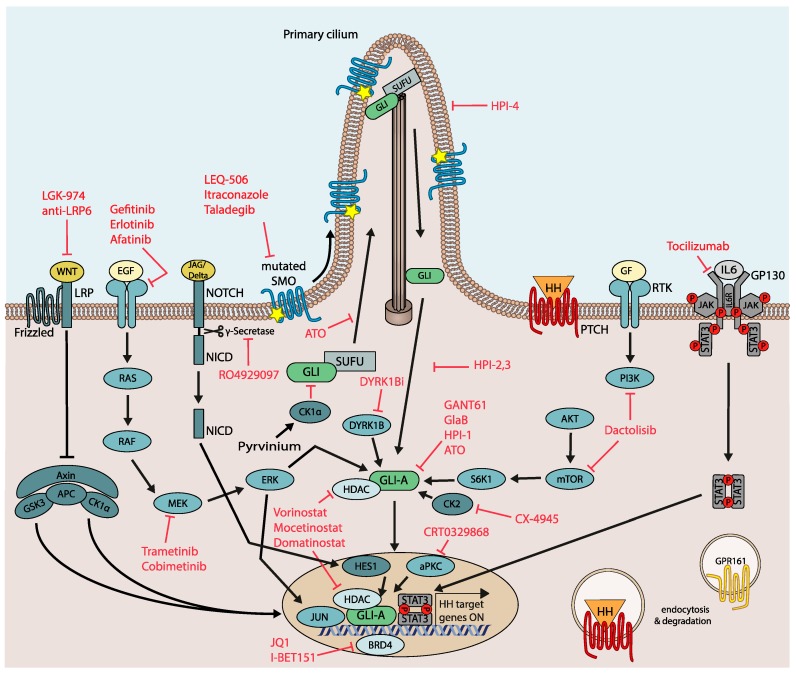
Therapeutic targeting of oncogenic HH/GLI signaling in cancer cells resistant to SMOi. Summary of targeting options to block pathways and signaling mechanisms accounting for SMO-independent GLI activation and SMOi resistance development. GF: Growth factor.

LEQ-506 is another SMO inhibitor able to inhibit HH signaling in the SMO^D473H^ medulloblastoma cell line derived from a patient that had relapsed after an initial response to vismodegib treatment [125] (Figure 3).

TAK-441 was discovered as a SMO antagonist in 2012 by Ohashi et al. [126]. The compound inhibits medulloblastoma growth in mouse allografts as well as disease progression and HH pathway activity in prostate cancer xenografts [126,127]. Furthermore, the drug shows an equal affinity for wild-type and SMO^D473H^, suggesting that it is a promising candidate for vismodegib-resistant HH-driven cancers [128]. However, no clinical trials with TAK-441 are currently ongoing (source: https://clinicaltrials.gov).

Itraconazole, a potent systemic antifungal drug, has also been identified as a potent HH/GLI antagonist by directly inhibiting SMO via a mechanism distinct from other SMO antagonists [129]. In contrast to vismodegib, itraconazole was able to inhibit murine SMO^D477G^ in vitro to a residual activity of ~30% and could inhibit proliferation of medulloblastoma tumor spheres expressing SMO^D477G^ to the same extent as SMO^WT^ tumor spheres [130]. In a mouse model of SMOi-resistant brain cancer, itraconazole administration in combination with the GLI antagonist arsenic trioxide (ATO) [131,132] efficiently overcame SMOi resistance and reduced tumor development [130]. However, the pronounced interaction of itraconazole with numerous other drugs poses significant limitations to its clinical use in oncology. A possibly more promising compound is the antifungal drug posaconazole, which exerts minimal drug–drug interactions, while potently inhibiting HH/GLI and drug-resistant SMO mutants in vitro and in vivo [133] (Figure 3). 

The novel SMO inhibitor taladegib (LY2940680) has been shown to reduce proliferation of HH-driven murine medulloblastoma as well as tumor growth of subcutaneous xenografts and HH pathway activity in the xenograft tumor stroma [134]. As the interaction of taladegib with amino acid 473 of SMO is less critical when compared to vismodegib [135], taladegib also binds to and inhibits vismodegib-resistant SMO^D473H^. Taladegib has already been under clinical investigation. In a phase I study including patients with naïve as well as previously treated BCC, taladegib drastically reduced GLI1 expression in skin biopsies and led to profound clinical response rates in both patient groups at drug doses that were well tolerated [136] (Figure 3).

### 6.2. Direct Targeting of Oncogenic GLI Transcription Factors

In light of the crucial function of GLI transcription factors in mediating the oncogenic activity of HH signaling, targeting GLI proteins is considered a promising yet challenging therapeutic strategy for HH-driven tumors. GANT61 represented the first GLI antagonist that may inhibit GLI proteins by interfering with their DNA binding capacity [137]. To date, no clinical studies with GANT61 have been done due to its restricted pharmacological suitability. 

Another direct GLI antagonist is ATO, a compound originally approved by the FDA for the treatment of acute promyelocytic leukemia [138]. ATO has been shown to inhibit GLI proteins via multiple modes, including the prevention of their ciliary activation, enhancement of degradation [131], and interference with GLI activity by direct binding to GLI proteins [132]. As mentioned above, ATO in combination with the SMO inhibitor itraconazole effectively overcomes SMO inhibitor resistance in models of medulloblastoma and BCC [130]. Another direct GLI inhibitor, glabrescione B (GlaB), an isoflavone naturally found in the seeds of *Derris glabrescens,* binds to GLI1 in its zinc finger domain, thereby diminishing GLI1/DNA interaction [139].

The Hedgehog pathway inhibitors (HPIs) HPI-1, HPI-2, HPI-3 and HPI-4 each inhibit GLI activity downstream of SUFU via unique mechanisms of action that have not yet been completely unraveled. HPI-1 is thought to target GLI1/2 independent of the ciliary machinery, HPI-2 and HPI-3 apparently inhibit GLI2 activation within the primary cilium, and HPI-4 disrupts the formation of the primary cilium itself [140]. After enhancing its aqueous solubility and bioavailability via encapsulation in polymeric nanoparticles, HPI-1 was given to mice harboring SMO^D477G^ medulloblastoma allografts and could significantly decrease tumor growth, as well as HH target gene expression [141] (Figure 3).

### 6.3. Targeting Modifiers of Oncogenic GLI Activity

As described in the previous chapter, resistance to SMO inhibitors can be initiated by the upregulation of GLI activity via molecular interactions and post-translational modifications. To circumvent the difficulties arising with the development of specific direct GLI inhibitors [142], recent strategies have focused on pharmacologically targetable molecules that had been shown to modulate GLI activity, involving kinases and epigenetic modifiers (Figure 3).

Targeting GLI modulating kinases offers a variety of therapeutic opportunities for the treatment of SMOi-resistant cancers. For instance, dactolisib, a dual PI3K and mTOR1/2 pathway inhibitor [143] with excellent clinical suitability [144], has shown efficacy in HH-driven medulloblastoma and SMOi-resistant chronic lymphocytic leukemia (CLL) alone or in combination with HH/GLI antagonists [145]. In medulloblastoma, dactolisib led to a decrease in PI3K-mTOR as well as HH pathway activity and inhibited proliferation of MB cell lines. Furthermore, a combination of vismodegib and dactolisib could significantly enhance the effect of single treatments to decrease MB tumor growth in NSG xenografts at drug concentrations that are clinically tolerable [146]. In CLL, PI3K/AKT and GLI activity have been shown to cooperatively promote the survival of leukemic cells. Consistently, combined targeting of PI3K and GLI, but not of SMO, led to a marked increase in apoptosis in a fraction of primary CLL patient samples [145].

As mentioned above, upregulated RAS/MAPK signaling is a frequent mechanism of SMO-independent GLI regulation and acquired drug resistance to SMO inhibitors, particularly in medulloblastomas and BCC. In agreement with these findings, administration of MEK inhibitors decreased both HH/GLI target gene expression and survival of SMOi-resistant cancer cells, suggesting therapeutic use of MEK inhibitors for MB and BCC patients with resistance-inducing SMO mutants or as a strategy to delay the onset of SMOi resistance [92,93]. The involvement of MEK/ERK in GLI regulation has further been shown in other malignant entities, such as melanoma and prostate cancer, suggesting that a broader applicability of MEK inhibitor treatment may be more generally efficacious in GLI-associated malignancies [95].

Members of the DYRK-family of protein kinases modulate GLI activity. Genetic inhibition of DYRK1B has been shown to reduce HH/GLI pathway activity in vismodegib-resistant tumor models and a novel small molecule DYRK1B inhibitor reduced in vitro tumorsphere and in vivo tumor growth of SMO-independent yet GLI-dependent cancer cells [94].

Aside from kinase inhibitors reducing GLI activity, epigenetic drugs provide another promising strategy to overcome SMOi resistance. Drug targeting of HDAC family members negatively affects GLI1/2 transcriptional activity by blocking deacetylation of GLI protein. In line with the GLI activity promoting role of HDACs, treatment with the selective HDAC1/2 inhibitor mocetinostat drastically reduced tumor growth in medulloblastoma mouse models [147]. Further, the clinically suitable class I HDAC inhibitor domatinostat (4SC-202) [148] abrogated GLI activity in cellular models resistant to SMO inhibitors [149]. Along this line, combined inhibition of HDAC1 and the GLI activator aPKC in SMO inhibitor resistant BCC models resulted in a synergistically improved in vivo response and therapeutic efficacy [150].

In addition to epigenetic drugs targeting HDACs, BRD4 inhibitors such as JQ1 are promising compounds in SMOi-resistant settings, alone or in combination with GLI antagonists. BRD4 inhibitor treatment led to a substantial decrease of the BRD4-GLI1/2 promoter interactions, and genes with GLI1 binding sites were downregulated after JQ1 treatment. HH-driven BCC and MB were sensitive to JQ1 treatment even in the presence of mutations that led to SMO inhibitor resistance [151]. I-BET151 was discovered as a small molecule BET protein inhibitor that inhibits HH target gene expression in Smo-independent *Sufu^−/−^* mouse embryonic fibroblasts and was able to reverse the interaction of Brd4 with the *Gli1* promoter. I-BET151 suppressed MB cancer stem cell viability in vitro and HH-dependent MB tumor growth in vivo [110]. Targeting BRD4 with clinically suitable drugs either alone or in combination with SMO/GLI antagonists is, therefore, a rational combination approach for the treatment of HH/GLI-associated malignancies.

SRF, a noncanonical GLI activator in SMO inhibitor resistant BCC, is activated via the formation of a heterodimeric complex with MKL1. The SRF/MKL1 pathway inhibitor CCG-1423 was able to inhibit cell growth and *Gli1* expression in SMOi-resistant murine BCC cell lines. Notably, this effect could be confirmed in human BCC [111].

### 6.4. Rational Combination Therapies Targeting Signal Integration and Cooperation—Future Applications of WNT, EGFR and IL6 Pathway Antagonists

The causal role of oncogenic WNT signaling in the context of HH/GLI-driven cancers is well established [152,153]. As already described in more detail above, insight into the mechanisms of acquired resistance in BCC uncovered a central role of WNT signaling in the lack of response to SMOi. In murine as well as human BCC, LGR5^+^ tumor cells were shown to be responsible for tumor relapse after initial response to SMOi treatment. Dual inhibition of WNT and HH/GLI signaling by a combination of vismodegib and the WNT inhibitor LGK-974 strongly decreased tumor burden in a murine BCC model, much more efficiently than either inhibitor alone. Intriguingly, only the combination treatment was able to block tumor relapse after treatment discontinuation, which was accompanied by a quantitative elimination of LGR5^+^ lesions during treatment [119]. Another combinatorial strategy using an anti-LRP6 antibody together with vismodegib efficiently decreased the number of residual murine BCC tumor nests and delayed tumor regrowth after treatment discontinuation [120]. 

Synergistic signal integration of, for instance, HH/GLI and the EGFR or IL6/JAK/STAT signaling pathway contribute to tumor growth and, therefore, offer additional opportunities for rational combination therapies. Indeed, pharmacologic EGFR targeting using clinically approved tyrosine kinase inhibitors or inhibition of common HH-EGFR target genes proved efficient in preclinical models by interfering with the growth and development of HH/GLI associated cancers and cancer stem cells [96,115,116,117]. 

## 7. Concluding Remarks

The HH/GLI signaling pathway is a highly complex network that plays a causal oncogenic role in several cancer entities, particularly in rare yet highly malignant CSC. The successful development of HH/GLI pathway inhibitors has mainly focused on targeting the essential HH/GLI effector SMO. Despite showing impressive therapeutic activity in nonmelanoma skin cancer, brain cancer, and AML, frequent and rapid resistance development urgently calls for the next generation of HH pathway inhibitors able to block both wild-type and mutant SMO proteins. Although this approach is key to more durable therapeutic effects for BCC, MB and most likely also AML patients, SMO-independent GLI activation remains a major limitation for the successful use of SMO antagonists that needs to be overcome for the therapy of a number of malignancies with high medical need. In this review, we have summarized druggable GLI modifiers that contribute to the oncogenic properties of HH/GLI-independent SMO activation. These regulatory interactions open up a broad field for the clinical evaluation of rational combination treatments to successfully control and interfere with the genetic and epigenetic dynamics of tumor development conferring resistance to temporarily successful monotherapies. Finally, we also envision that HH/GLI pathway blockers in combination with drugs targeting immune evasion mechanisms, such as immune checkpoint inhibitors, are likely to provide further therapeutic benefit. Although our present knowledge about the impact of HH/GLI on tumor immune evasion is fairly limited, first evidence from preclinical and clinical observations suggests that next-generation HH/GLI inhibitors together with strategies reactivating the antitumoral immune response may represent the next leap for anti-HH/GLI therapy of cancers [154,155,156,157,158].
